# Phase I trial of pod-intravaginal rings delivering antiretroviral agents for HIV-1 prevention: Rectal drug exposure from vaginal dosing with tenofovir disoproxil fumarate, emtricitabine, and maraviroc

**DOI:** 10.1371/journal.pone.0201952

**Published:** 2018-08-22

**Authors:** Kathleen Listiak Vincent, John A. Moss, Mark A. Marzinke, Craig W. Hendrix, Peter A. Anton, Manjula Gunawardana, Lauren N. Dawson, Trevelyn J. Olive, Richard B. Pyles, Marc M. Baum

**Affiliations:** 1 Department of Obstetrics and Gynecology, University of Texas Medical Branch, Galveston, Texas, United States of America; 2 Department of Chemistry, Oak Crest Institute of Science, Monrovia, California, United States of America; 3 Department of Medicine (Division of Clinical Pharmacology), Johns Hopkins University, Baltimore, Maryland, United States of America; 4 Department of Pathology, Johns Hopkins University, Sheikh Zayed Tower, Baltimore, Maryland, United States of America; 5 Center for HIV Prevention Research, Division of Digestive Diseases and UCLA AIDS Institute, David Geffen School of Medicine, University of California Los Angeles, Los Angeles, California, United States of America; 6 Department of Pediatrics, University of Texas Medical Branch, Galveston, Texas, United States of America; 7 Department of Microbiology and Immunology, University of Texas Medical Branch, Galveston, Texas, United States of America; Chiang Mai University, THAILAND

## Abstract

**Background:**

Intravaginal rings (IVRs) can deliver antiretroviral (ARV) agents for HIV pre-exposure prophylaxis (PrEP), theoretically overcoming adherence concerns associated with frequent dosing. However, topical vaginal ARV drug delivery has not simultaneously led to sufficient rectal drug exposure to likely protect from HIV infection as a result of receptive anal intercourse (RAI). Unprotected RAI has a higher risk of infection per sex act and, for women, also can be associated with vaginal exposure during a single sexual encounter, especially in higher-risk subsets of women. The physiologically inflamed, activated, immune-cell dense colorectal mucosa is increasingly appreciated as the sexual compartment with highly significant risk; this risk is increased in the setting of co-infections. *Ex vivo* studies have shown that colorectal tissue and rectal fluid concentrations correlated with HIV protection. Given these important results, efforts to document colorectal compartment ARV drug concentration from pod-IVR delivery was assessed to determine if vaginal application could provide protective ARV levels in both compartments.

**Methodology/Principal findings:**

A crossover clinical trial (*N* = 6) evaluated 7 d of continuous TDF pod-IVR use, a wash-out phase, followed by 7 d with a TDF-FTC pod-IVR. A subsequent clinical trial (*N* = 6) consisted of 7 d of continuous TDF-FTC-MVC pod-IVR use. Rectal fluids were collected on Day 7 at IVR removal in all three ARV-exposures (two Phase 1 trials) and drug concentrations quantified by LC-MS/MS.

Median rectal fluid concentrations of TFV, the hydrolysis product of the prodrug TDF, were between 0.66 ng mg^-1^ (TDF pod-IVR group) and 1.11 ng mg^-1^ (TDF-FTC pod-IVR group), but below the analytical lower limit of quantitation in 5/6 samples in the TDF-FTC-MVC pod-IVR group. Unexpectedly, median FTC (TDF-FTC pod-IVR, 20.3 ng mg^-1^; TDF-FTC-MVC pod-IVR, 0.18 ng mg^-1^), and MVC rectal fluid concentrations (0.84 ng mg^-1^) were quantifiable and higher than their respective *in vitro* EC_50_ values in most samples. Due to participant burden in these exploratory trials, rectal fluid was used as a surrogate for rectal tissue, where drug concentrations are expected to be higher.

**Conclusions/Significance:**

The concentrations of FTC and MVC in rectal fluids obtained in two exploratory clinical trials of IVRs delivering ARV combinations exceeded levels associated with *in vitro* efficacy in HIV inhibition. Unexpectedly, MVC appeared to depress the distribution of TFV and FTC into the rectal lumen. Here we show that vaginal delivery of ARV combinations may provide adherence and coitally independent dual-compartment protection from HIV infection during both vaginal and receptive anal intercourse.

## Introduction

Topical dosing of antiretroviral (ARV) agents using products formulated for rectal or vaginal application have the potential of protecting against sexually transmitted infections, including HIV. Intravaginal rings (IVRs) can deliver antiretroviral (ARV) agents for HIV pre-exposure prophylaxis (PrEP) [1–3], theoretically overcoming adherence concerns associated with frequent dosing [4]. We have developed an innovative IVR technology, the pod-IVR [1, 5], that enables accelerated development of products capable of delivering multiple agents over a wide range of aqueous solubilities and target delivery rates into the cervicovaginal compartment [2, 3, 6–8].

We have evaluated the clinical pharmacokinetics (PKs) and safety of pod-IVRs delivering the nucleoside reverse transcriptase inhibitor (NRTI) tenofovir disoproxil fumarate (TDF) alone and in combination with emtricitabine (FTC) [9], another NRTI. We subsequently conducted a clinical trial with the first triple combination ARV pod-IVR simultaneously delivering TDF, FTC, and maraviroc (MVC), an inhibitor/antagonist of chemokine receptor CCR5 [9].

Topical vaginal ARV drug delivery has not been well-evaluated to determine if this route of administration could lead to simultaneous rectal drug exposure sufficient to protect from HIV infection as a result of receptive anal intercourse (RAI) [10–14]. Exposure through RAI has a higher risk of infection per sex act and, for women, especially those in higher-risk subsets, there is the possibility of both vaginal and rectal exposure during a single sex act [15–17]. The physiologically inflamed, activated, immune-cell dense colorectal mucosa is increasingly appreciated as the sexual compartment with highly significant risk. *Ex vivo* HIV challenge of colorectal biopsies from humans receiving oral, vaginal, and rectal ARV drugs have demonstrated suppression of HIV infection, sometimes with clear concentration-response relationships [18–26].

The risk of HIV acquisition associated with both vaginal and rectal sexual intercourse, along with the above results informing potential efficacious ARV drug concentrations needed for rectal protection, have led us to collect rectal fluids in the above clinical trials evaluating pod-IVRs delivering TDF, TDF-FTC, and TDF-FTC-MVC. Colorectal compartment drug concentrations are assessed here to determine if vaginal application could simultaneously provide protective ARV levels in both compartments.

## Materials and methods

### Ethics statement

All human research was approved by the University of Texas Medical Branch Institutional Review Board (IRB # 14–0479), conducted according to the Declaration of Helsinki, and registered in clinicaltrials.gov (NCT02431273; https://clinicaltrials.gov/ct2/show/NCT02431273?term=NCT02431273&rank=1). All participants provided written informed consent.

### Clinical trial design

The early Phase I clinical studies were performed between June 2015 and July 2016 at the Clinical Research Center of the University of Texas Medical Branch in Galveston, Texas. Women were recruited by announcements and word of mouth and underwent a phone pre-screen prior to attending a screening visit where informed consent was obtained and inclusion/exclusion criteria were confirmed. Inclusion criteria included age 18–45, regular menstrual cycles, use of contraception, and agreement to abstain from sexual intercourse during use of the IVR until the follow up exam 1–2 weeks after discontinuation of IVR use. Exclusion criteria included HIV, Heptatitis B, or sexually transmitted disease (gonorrhea, chlamydia, trichomonas) at the time of screening, abnormal liver or kidney function tests, and pregnancy/lactation.

**Fig 1:**
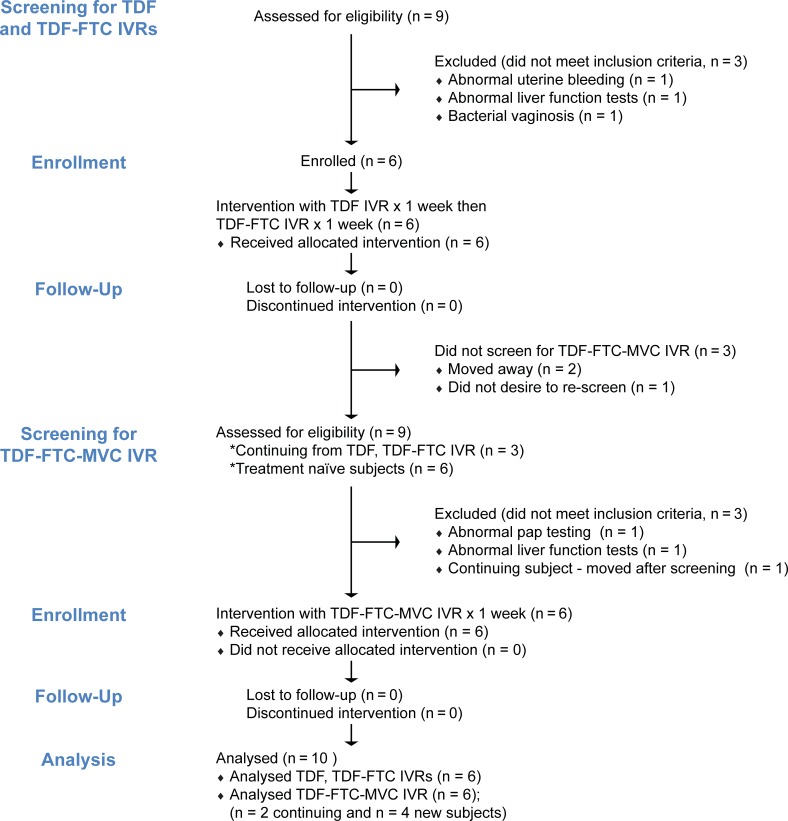
Participant flow diagram.

Women initially participated in a clinical trial where they used a pod-IVR releasing TDF and a TDF-FTC combination for 7 days in a cross-over, open label design, with at least 2 weeks of washout between IVRs (*n* = 6) (Fig 1) [9]. After the study team ensured that the IVRs were safe and provided adequate drug release, the women were invited to re-screen for a continuation of the study where they used a pod-IVR releasing the TDF-FTC-MVC combination for 7 days in an open label design, with new subjects additionally recruited in order to enroll a total of 6 women to use this IVR (Fig 1) [9]. In all three arms, vaginal fluid (Dacron swab), vaginal biopsy, and rectal fluid (Dacron swab) samples were collected on Day 7, immediately after IVR removal. Rectal biopsies were not acquired during these studies due to the increased participant burden. [20]

### Chemicals, reagents, and fabrication of pod-IVRs

Materials and methods related to the IVR fabrication have been provided elsewhere [9] Briefly, TDF and FTC (labeled for human use) were purchased from commercial vendors with a Drug Master File (DMF) registered with the FDA. MVC was isolated from the commercial formulation (Pfizer, Inc., New York, NY), which consists of film-coated tablets for oral administration containing 300 mg of MVC and inactive ingredients, as described previously [2]. All other reagents were obtained from Sigma-Aldrich, unless otherwise noted.

Polydimethylsiloxane (PDMS, silicone) pod-IVRs were fabricated has according to methods described in detail elsewhere [2, 3, 5, 27]. The IVR drug content was as follows: TDF, 180 mg; FTC, 135 mg; MVC, 90 mg. The amount of residual drug remaining in used IVRs was measured by HPLC according to published methods [2, 27].

### Bioanalysis of *in vivo* samples

Concentrations of TDF, TFV, TFV-DP, FTC, and MVC in fluids/tissue were determined *via* previously described liquid chromatographic-tandem mass spectrometric (LC-MS/MS) assays [11, 28–30]. All assays were developed and validated following the Food and Drug Administration Guidance for Industry, Bioanalytical Method Validation recommendations and met all acceptability criteria [31]. Isotopically labeled internal standards were used for all compounds and the determination of drug concentrations in all specimen sources.

The lower limits of quantification for these methods were as follows: cervicovaginal fluids (CVFs), TDF (0.0625 ng/sample), TFV (0.25 ng/sample), FTC (1.0 ng/sample), MVC (0.05 ng/sample); vaginal tissue homogenate, TFV (0.05 ng/sample), FTC (0.25 ng/sample), MVC (0.05 ng/sample); rectal fluids, TFV (0.25 ng/sample), FTC (1.0 ng/sample), MVC (0.05 ng/sample). Drug concentrations in luminal fluid and tissue samples were ultimately reported as ng mg^-1^ or fmol mg^-1^, respectively, following normalization to net biopsy or Dacron swab weight. Post-dose concentrations below the corresponding LLOQs (*C*_*LLQ*_) were imputed as follows in all analyses:

$$C_{\mathrm{LLQ}}=\frac{\mathrm{LLOQ}\;\mathrm{of}\;\mathrm{assay}}{2\times (\mathrm{median}\;\mathrm{sample}\;\mathrm{mass})}$$

Results included here to provide intra-subject and between-group context for interpreting the comparative utility of rectal fluid results necessarily include CVF and VT concentration data from these trials that was also published separately [9].

### Statistical analysis

Data were analyzed using GraphPad Prism (version 7.00, GraphPad Software, Inc., La Jolla, CA). Statistical significance is defined as *P* < 0.05.

## Results

Details on IVR safety, drug concentrations in vaginal fluid and tissue, and user perception have been provided elsewhere [9]. In brief, no significant AEs or behavioral concerns were identified with use of any of the pod-IVRs. All participants completed all study visits and there were no missed visits, drop-outs, or loss to follow-up (Fig 1).

### Drug concentration measurements

**Fig 2:**
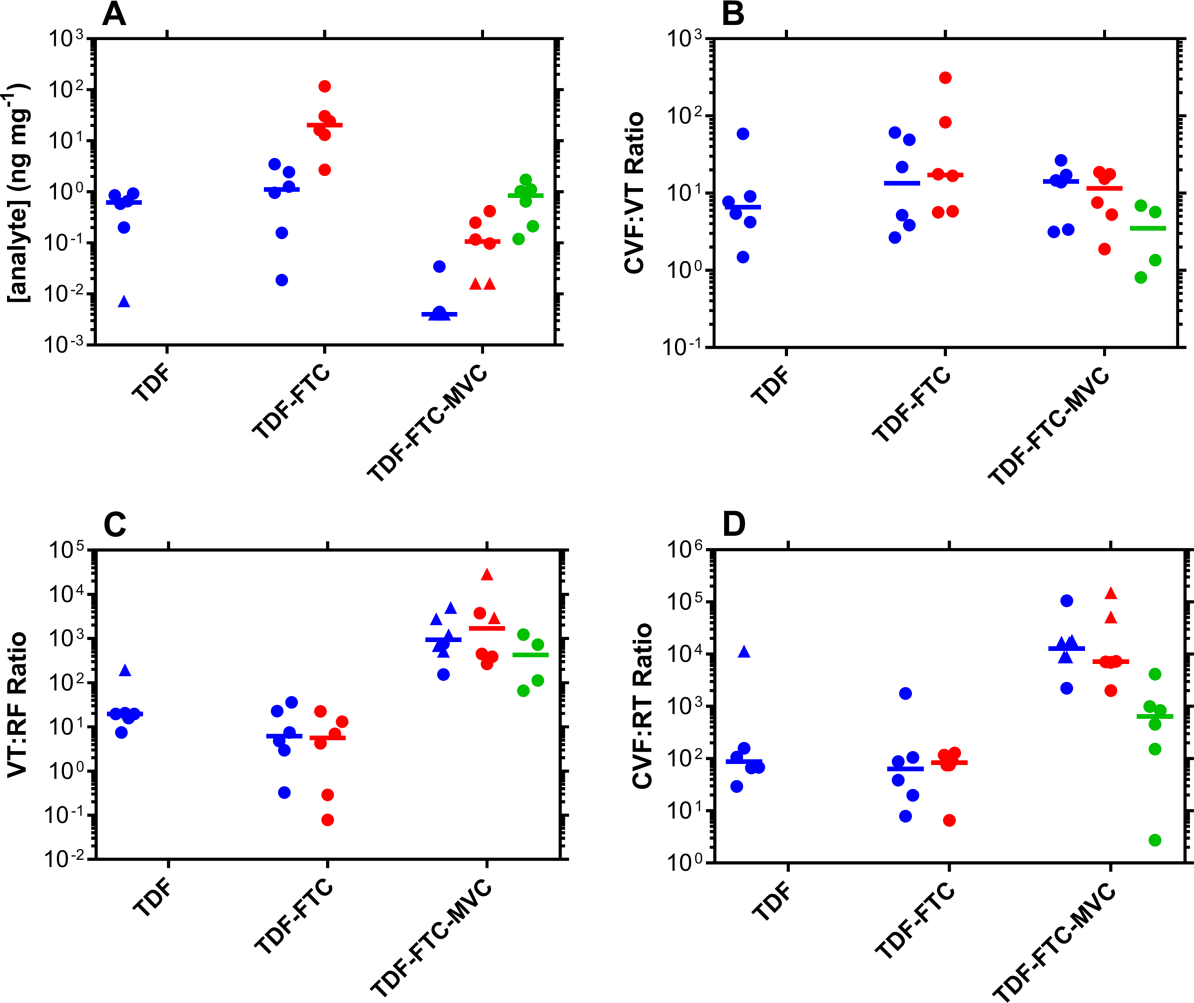
Rectal fluid ARV drug exposure and distribution following IVR delivery. Every circular datum represents an individual sample from one of the participants (*n* = 6); horizontal lines represent group medians; blue, TFV; red, FTC; green, MVC. (A) rectal fluid drug concentrations on Day 7. Paired Day 7 concentration ratios across anatomic compartments provide a measure of drug distribution; (B) cervicovaginal fluid to vaginal tissue (CVF:VT), with two VT MVC samples omitted as these were ALQ; (C) vaginal tissue to rectal fluid (VT:RF); (D) cervicovaginal fluid to rectal fluid (CVF:RF). Triangles represent samples where the rectal fluid drug concentration was BLQ.

Drug concentrations in key anatomic compartments on Day 7 are summarized in Table 1 and Fig 2A. All Rectal fluid TFV concentrations resulting from either the TDF or the TDF-FTC pod-IVRs were not significantly different (*P* = 0.20), using a two-tailed paired *t* test. While important on its own, rectal fluid TFV concentrations in the TDF-FTC-MVC group was quantifiable in only one out of six samples (Table 1). Rectal fluid FTC concentrations resulting from either the TDF-FTC or the TDF-FTC-MVC pod-IVRs were not significantly different (*P* = 0.10), using an unpaired *t* test with Welch’s correction (i.e., do not assume equal standard deviations). While TFV concentrations in rectal fluid were similar in the TDF (median, 0.62 ng mg^-1^; IQR, 0.30–0.81 ng mg^-1^) and TDF-FTC (median, 1.11 ng mg^-1^; IQR, 0.36–2.15 ng mg^-1^) groups, median FTC concentrations in rectal fluid in the TDF-FTC (median, 20.3 ng mg^-1^; IQR, 14.0–28.9 ng mg^-1^) group were 190 times higher than in the TDF-FTC-MVC (median, 0.11 ng mg^-1^; IQR, 0.04–0.22 ng mg^-1^) group.

**Table 1:** Summary of drug concentrations in key anatomic compartments on Day 7, immediately after pod-IVR removal (six participants in each group). CVF, cervicovaginal fluid; VT, vaginal tissue; RF, rectal fluid. Measurements outside of the analytical ranges were not included in the analysis.

	Pod-IVR Type^a^		
Analyte, matrix	TDF	TDF-FTC	TDF-FTC-MVC
Total TFV,^b^ CVF, ng mg^-1^	51.3 (34.8–61.5)	40.8 (28.9–49.1)	67.4 (43.4–72.1)
FTC, CVF, ng mg^-1^		1,458 (881–2,073)	839 (818–1,572)
MVC, CVF, ng mg^-1^			367 (224–489)
TFV, VT ^c^, ng mg^-1^	8.4 (4.7–11.2)	5.1 (0.77–10.1)	5.1 (3.3–9.7)
FTC, VT ^c^, ng mg^-1^		74.5 (11.6–193)	104 (63.7–302)
MVC, VT ^c^, ng mg^-1^			142 (82.5–212)^d^
TFV, RF, ng mg^-1^	0.62 (0.30–0.81)^e^	1.11 (0.36–2.15)	0.004^f^
FTC, RF, ng mg^-1^		20.3 (14.0–28.9)	0.11 (0.04–0.22)^g^
MVC, RF, ng mg^-1^			0.84 (0.32–1.10)

^a^Median (Interquartile range, 25th to 75th percentile).

^b^Molar sum of paired TDF and TFV concentrations, reported as TFV.

^c^Used with permission [9]

^d^33% of samples above the analytical quantification limit.

^e^83% of samples were above the analytical LLQ.

^f^Only one sample was above the analytical LLQ.

^g^67% of samples were above the analytical LLQ.

The collection and analysis of cervicovaginal fluid (CVF), vaginal tissue (VT), and rectal fluid (RF) samples from all six study participants allows paired drug concentration ratios (i.e., CVF:VT, VT:RF, and CVF:RF) to be compared across all three compartments in Table 2 and Fig 2B and 2C. The TFV CVF:VT ratios across all three groups were compared with a nonparametric, Kruskal-Wallis test and determined not to be significantly different (*P* = 0.85). The FTC CVF:VT ratios in the TDF-FTC and TDF-FTC-MVC pod-IVR groups also were not significantly different (*P* = 0.26), using an unpaired *t* test with Welch’s correction.

**Table 2:** Paired drug concentration ratios by anatomic compartment on Day 7, immediately after pod-IVR removal (six participants in each group). CVF, cervicovaginal fluid; VT, vaginal tissue; RF, rectal fluid. Data consist of medians (interquartile range) and only measurements within the analytical range of the assay are included.

	Pod-IVR Type		
Analyte, Ratio	TDF	TDF-FTC	TDF-FTC-MVC
TFV,^a^ CVF:VT	6.6 (4.5–8.8)	14 (4.2–42)	14 (6.0–17)
FTC, CVF:VT		17 (8.6–67)	12 (5.8–17)
MVC, CVF:VT			3.5 (1.2–6.0)
TFV, VT:RF	20 (17–20)	6.2 (3.4–19)	946 (559–2,396)
FTC, VT:RF		5.6 (1.3–12)	1,702 (404–3,565)
MVC, VT:RF			423 (101–855)
TFV,^a^ CVF:RF	87 (67–145)	63 (25–100)	12,763 (8,835–16,813)
FTC, CVF:RF		83 (75–111)	7,181 (6,978–40,726)
MVC, CVF:RF			644 (228–950)

^a^Molar sum of paired TDF and TFV concentrations in CVF, reported as TFV.

Tenofovir VT:RF ratios in the TDF (median, 20; IQR, 17–20) group were numerically 3.2 times higher than in the TDF-FTC (median, 6.2; IQR, 3.4–19) group, and 47 times lower than in the TDF-FTC-MVC (median, 946; IQR, 559–2,396) group, where most measurements were BLQ. Emtricitabine VT:RF ratios in the TDF-FTC (median, 5.6; IQR, 1.3–12) group were 304 times lower overall than in the TDF-FTC-MVC (median, 1,702; IQR, 404–3,565) group. However, with the small sample size, the VT:RF ratios were not significantly different for TFV (*P* = 0.31) in the TDF and TDF-FTC pod-IVR groups and for FTC (*P* = 0.24) in the TDF-FTC and TDF-FTC-MVC pod-IVR groups, using an unpaired *t* test with Welch’s correction.

## Discussion

Antiretroviral drug concentrations in rectal fluids were higher than expected following pod-IVR (i.e., vaginal) drug delivery in all three clinical trial arms. Tenofovir RF concentrations in the TDF (median, 0.62 ng mg^-1^; IQR, 0.30–0.81 ng mg^-1^) and TDF-FTC groups (median, 1.11 ng mg^-1^; IQR, 0.36–1.25 ng mg^-1^) were comparable to those reported on Day 7 in another clinical trial (median, 0.44 ng mg^-1^; IQR, 0.22–1.94 ng mg^-1^) evaluating a reservoir TDF IVR [14]. Our Day 7 TFV CVF:RF concentration ratios (Table 2, Fig 1D) in the TDF (median, 87; IQR, 67–145) and TDF-FTC groups (median, 63; IQR, 25–100) were lower than the corresponding ratios measured on Day 7 (median, 104; IQR, 59–505) in the clinical study involving the reservoir TDF IVR [14]. The lowest rectal fluid drug exposure was obtained with TFV, especially in the TDF-FTC-MVC group where only one of six samples was above the lower limit of quantitation of the assay.

In MTN-001, the pharmacokinetics of a 1% TFV (note, not TDF) vaginal gel were evaluated in a randomized, cross-over study involving 144 HIV-uninfected women, of which a subset of 12 had rectal sampling [11]. Comparing the end-of-period visit measurements of median CVF (3.1×10^3^ ng mg^-1^, estimated from CVL samples assuming a 20-fold dilution of the CVF) and RF (6.0 ng mg^-1^, estimated from rectal sponge measurements reported in ng/sponge and a median mass of rectal fluid collected of 20 mg) concentrations affords a median CFV:RF TFV concentration ratio of 517. In MTN-014, 14 HIV-uninfected women received daily vaginal TFV 1% (reduced glycerin formulation) gel for 2 weeks. The median TFV concentration in CVF swabs (7,138 ng/swab) and RF swabs (4.4 ng/swab) indicate a CVF:RF TFV ratio of ca. 1,622 [13]. Taken together, these two TFV vaginal gel dosing studies report CVF:RF TFV ratios roughly 10–25 times higher than those measured with our pod-IVR. The lower the CFV:RF concentration ratio, the more efficiently the drug is thought to partition from the vaginal to rectal lumen. Clinical data on vaginal to rectal drug distribution for FTC and MVC are not available outside of the current report.

The RF ARV exposure measured here following vaginal drug delivery may hold important implications for HIV PrEP efficacy following receptive anal intercourse (RAI). While EC_50_, or EC_90_, drug concentrations are not used here as target concentrations for HIV prevention, they provide a preliminary guide to gauge possible efficacy. The *in vitro* EC_50_ of TFV against HIV-1 was 0.18 μM in peripheral blood mononuclear cells (PBMCs) [32] and 0.5 μM in MT-2 cells [33]. Median *in vivo* TFV RF concentrations in the TDF and TDF-FTC pod-IVR groups were ca. 3.5 μM (1 ng mg^-1^), in the range currently thought to be needed to provide some level of protection from HIV infection. Median FTC RF concentrations in the TDF-FTC pod-IVR group (20 ng mg^-1^, 81 μM) and in the TDF-FTC-MVC pod-IVR group (0.11 ng mg^-1^, 0.43 μM) compare favorably to the range of EC_50_ (1×10^−3^–0.64 μM) and EC_90_ concentrations (0.06–0.35 μM) against HIV reported for a variety of *in vitro* assays [34–38]. Median *in vivo* MVC RF concentrations (0.84 ng mg^-1^, 1.6 μM) measured here were conservatively higher than the *in vitro* anti-HIV EC_50_ (0.1×10^−3^–6×10^−3^ μM) and EC_90_ concentrations (0.5×10^−3^–0.013 μM) reported in the literature [37, 39].

The above preliminary analysis suggests that dual compartment protection from HIV infection may be possible with combination pod-IVRs, with the following important caveats. First, adding MVC to the TDF-FTC combination was associated with a decrease in measurable levels of TFV and FTC in rectal fluids (Fig 2A), despite similar concentrations of TDF/TFV and FTC across groups in CFV and VT (Table 1). While these two drugs work intracellularly in target cells, underscoring the importance of direct tissue measurements, the underlying mechanism for TFV and FTC RF reductions in the presence of vaginally-administered MVC is unknown, but could involve changes in molecular transporter expression [40, 41], or transporter inhibition, in the rectovaginal septum (i.e., layer of tissue between the vagina and the rectum). Second, colorectal tissue, not the lumen, is the likely pharmacologic compartment determining HIV PrEP efficacy.

While ARV drug concentrations in rectal tissues were not measured here, the drug concentration ratios across compartments (Table 2, Fig 1B–1D) suggest that the agents distribute across the rectovaginal septum, rather first than through systemic circulation on the way to the rectal tissue. This hypothesis suggests that anterior (i.e., closer in proximity to the vagina) colorectal tissue drug concentrations will be higher than in rectal fluids, which in turn may exceed posterior colorectal tissue drug concentrations. It is theoretically possible that vaginal to rectal drug distribution could occur through a combination passive and active transport along with local venous and lymphatic drainage. The luminal and mucosal colorectal drug distribution is critical in determining potential efficacy in HIV prevention, as discussed in Weld *et al*. [42], and needs to be studied further in future pod-IVR trials. Topical dosing has the advantage of producing very high local drug concentrations that may be sufficient to achieve diffusion to a second compartment such that, even with a steep concentration gradient, a protective level might be obtained, especially if activation of the drug is different in the second compartment. For example, colon *versus* cervicovaginal intracellular phosphorylation differences could completely compensate for diffusion-dependent concentration gradients of the parent drug by producing more phosphorylated TFV or FTC in the second compartment.

In conclusion, rectal fluid ARV drug concentrations resulting from vaginal dosing using three different pod-IVR formulations were detectable and, higher than anticipated. The results suggest that dual compartment protection from sexual HIV infection may be possible while maintaining a low systemic drug exposure. These important findings warrant further investigation in larger clinical trials.

## Supporting information

S1 Fig TREND Statement checklist. (PDF)

S1 Text Protocol summary. (DOCX)

S1 Table Drug concentrations in rectal fluid samples collected on the day of TDF pod-IVR removal (six participants); i.e., Day 7. (DOCX)

S2 Table Drug concentrations in rectal fluid samples collected on the day of TDF-FTC pod-IVR removal (six participants); i.e., Day 7. (DOCX)

S3 Table Drug concentrations in rectal fluid samples collected on the day of TDF-FTC-MVC pod-IVR removal (six participants); i.e., Day 7. (DOCX)
