# Supplementary material for: Phase I trial of pod-intravaginal rings delivering antiretroviral agents for HIV-1 prevention: Rectal drug exposure from vaginal dosing with tenofovir disoproxil fumarate, emtricitabine, and maraviroc
Source: PLoS One. 2018 Aug 22;13(8):e0201952. doi: 10.1371/journal.pone.0201952 (PMC6104940; doi:10.1371/journal.pone.0201952)
Supplement: S1 Text — (DOCX) [file pone.0201952.s002.docx]

# PROTOCOL SUMMARY

Short Title: PrEP-Pod-IVR-01

Clinical Phase: Pre-Phase 1

IND Number: 123099

IND Sponsor: Auritec Pharmaceuticals, Inc

Principal Investigator: Kathleen L. Vincent, M.D.

Sample Size: 6 female participants

Study Population: Healthy HIV-negative, pre-menopausal females 18-45 years of age, who met the eligibility criteria were enrolled in this trial if they provided valid written informed consent.

Study Site: University of Texas Medical Branch at Galveston (Clinical)

The Miriam Hospital, Rhode Island (Remote Behavioral Site)

Study Duration: June 2015 - July 2016

Study Products: Silicone pod intravaginal ring (pod-IVR) containing pods (i.e., polymer-coated drug tablets) of the following:

- tenofovir disoproxil fumarate (TDF)
- tenofovir disoproxil fumarate (TDF)-emtricitabine (FTC)
- tenofovir disoproxil fumarate (TDF)-emtricitabine (FTC)-maraviroc (MVC)

Table 1: Study Regimen

| **Treatment Period 1** | **Washout Period** | **Treatment Period 2** | **Washout Period** | **Treatment Period 3** |
| --- | --- | --- | --- | --- |
| **7 days** | **≥14 days** | **7 days** | **≥14 days** | **7 days** |
| TDF Only IVR | No ring | TDF-FTC IVR | No ring | TDF-FTC-MVC IVR |

# OVERALL STUDY DESIGN

This was an open-label, Pre-Phase I study, conducted at a single research center in the USA among 6 healthy, human immunodeficiency virus (HIV)-uninfected, adult females, per treatment period to assess the safety, acceptability and pharmacokinetics of 3 different pod-IVRs delivering 3 different ARV regimens [2, 3].

Women who gave written informed consent were invited to screen for the trial and, if they were generally healthy and met specified inclusion/exclusion criteria, they could be enrolled in the trial.

There were 3 separate 7-day treatment periods (Treatment Period 1- TDF only pod-IVR, Treatment Period 2- TDF-FTC pod-IVR, and Treatment Period 3- TDF-FTC-MVC pod-IVR). Each participant used each pod-IVR for 7 days with a washout period of at least 14 days between each treatment period. Progression to the next treatment period was contingent upon the absence of grade 3 or 4 genitourinary adverse events considered to be drug-related by the investigator or other investigator assessed drug-related serious adverse event.

Participants had clinical safety assessments, including colposcopy and optical coherence tomography (OCT), and sample collection on the day of each IVR insertion (Visits 1, 5, and 9), 2 days after insertion (Visits 2, 6, and 10), and 7 days after insertion (Visits 3, 7, and 11), and 1-2 weeks after removal (Visits 4, 7, and 12). At Visits 3, 7, and 11 the IVR was removed and participants completed a computer-administered self-interview (CASI) regarding acceptability. Sample collection included blood and vaginal fluids at each visit and vaginal tissue biopsies and rectal swabs at the end of each treatment period (Visits 3, 7, and 11). At Visits 4, 8, and 12 also completed an in-depth interview.

Table 4. Study Design

|  |  | Treatment Period 1  TDF only pod-IVR | | | | Treatment Period 2  TDF-FTC pod-IVR | | | | Treatment Period 3  TDF-FTC-MVC pod-IVR | | | |
| --- | --- | --- | --- | --- | --- | --- | --- | --- | --- | --- | --- | --- | --- |
|  | V0 | V1 | V2 | V3 | V4 | V5 | V6 | V7 | V8 | V9 | V10 | V11 | V12 |
|  | Screening | D0 | D2 | D7 | D14-21 | D0 | D2 | D7 | D14-21 | D0 | D2 | D7 | D14-21 |
|  |  | Insertion |  | Removal |  | Insertion |  | Removal |  | Insertion |  | Removal |  |
| CBC, AST, ALT, Creatinine | X |  |  |  | X |  |  |  | X |  |  |  | X |
| STI Testing | X |  |  |  | X |  |  |  |  |  |  |  |  |
| HBsAg | X |  |  |  | X |  |  |  |  |  |  |  |  |
| HIV serology | X |  |  |  | X |  |  |  |  |  |  |  |  |
| hCG | X | X | X | X |  | X | X | X |  | X | X | X |  |
| Colposcopy | X | X | X | X | X | X | X | X | X | X | X | X | X |
| OCT |  | X | X | X | X | X | X | X | X | X | X | X | X |
| PK |  |  |  |  |  |  |  |  |  |  |  |  |  |
| Vaginal Fluid |  | X | X | X | X | X | X | X | X | X | X | X | X |
| CVL |  | X | X | X | X | X | X | X | X | X | X | X | X |
| Rectal Fluid |  |  |  | X |  |  |  | X |  |  |  | X |  |
| Vaginal Tissue |  |  |  | X |  |  |  | X |  |  |  | X |  |
| Inflammatory Markers | X | X | X | X | X | X | X | X | X | X | X | X | X |
| Cell Products | X | X | X | X | X | X | X | X | X | X | X | X | X |
| VMB | X | X | X | X | X | X | X | X | X | X | X | X | X |
| Histology |  |  |  | X |  |  |  | X |  |  |  | X |  |
| CASI | X |  |  | X |  |  |  | X |  |  |  | X |  |
| In-depth Interview |  |  |  |  | X |  |  |  | X |  |  |  | X |

* All participants also had a screening visit (V0) prior to Visit 9.

^Two completed all three treatment periods. Four participants were replaced after the second treatment period. The four replacement participants completed the third treatment period only (TDF-FTC-MVC pod-IVR).

# STUDY OBJECTIVES

- To determine the safety of Single (TDF), Dual (TDF-FTC), and Triple (TDF-FTC-MVC) pod-IVRs
- To determine pharmacokinetics of ARV IVRs by measuring the concentrations of each drug in plasma, vaginal fluid, cervicovaginal lavage, rectal fluid, and vaginal tissue
- To determine the acceptability and perceptibility of the pod-IVR design

# STUDY Endpoints:

**Safety**

- AEs as defined by the DAIDS AE Grading Table Version 2.0, November 2014, including Addendum 1 (Female Genital Toxicity Table for Use in Topical Microbicide Studies)

**Pharmacokinetics**

- TDF, FTC, and MVC drug, and drug metabolite, concentrations in
  - Plasma
  - Vaginal fluid
  - Cervicovaginal lavage (CVL; pellet and supernatant)
  - Rectal fluid
  - Vaginal tissue

**Mucosal Safety**

- Vaginal epithelial thickness measured by optical coherence tomography (OCT)
- Number of colposcopy findings
- Vaginal histology (tissue)
- Cytology via microscopy (CVL and IVR wash)
- Phenotyping by flow cytometry (CVL)
- Microbiology (e.g., pH, Nugent Score, VMB culture)
  - vaginal wall swabs (i.e., daily self-collected & study visit swabs)
  - CVL

**Perceptibility and Acceptability**

- Self-reported attitudes of participants about experience and product attributes via CASI and in-depth interviews

# Inclusion Criteria

1. Provides written informed consent
2. Healthy female 18-45 years of age
3. HIV negative per subject report and results of screening examination
4. Negative for sexually transmitted diseases in the past 3 months and at screening exam
5. Currently using contraception with plans to continue throughout the study duration or having sex with females only. Acceptable forms of contraception include oral, subcutaneous, subdermal, intramuscular, intrauterine or permanent/surgical methods.
6. Pre-menopausal with a regular menstrual cycle with at least 21 days between menses and no recent, untreated, history of intermenstrual bleeding or with suppressed menstrual cycle by hormonal contraception such as Depo-Provera or continuous oral contraceptive agents.
7. Subjects must agree to abstain from vaginal, anal, and oral sex throughout the first week of each dosing period and then use condoms (without N-9) for vaginal/rectal intercourse until after the final visit for use of each IVR
8. Subjects must agree to not douche or use any vaginal product other than the Single, Dual and Triple ARV IVRs, including lubricants, feminine hygiene products, and vaginal drying agents throughout the dosing period and until after the final visit
9. Subjects must agree to blood draws and vaginal exams throughout the course of the study

# Exclusion Criteria

1. HIV positive by subject report or results of screening examination
2. Positive history for autoimmune disease
3. Abnormal genital exam defined as grade 1 or higher adverse event by DAIDS genital AE grading table
4. Abnormal ALT or AST or Hepatitis B infection
5. Active genital herpes I or II infection
6. Active vaginal infection as determined by site IoR
7. Abnormal renal function (defined as a creatinine clearance of <50mL/min/1.73 m2)
8. Pregnant or less than 6 months post-partum or current lactation
9. Current use of an IVR (i.e., Nuvaring, Estring, Femring)
10. History of TDF, FTC, and MVC use and/or adverse reaction to any of these drugs
11. History of adverse reaction to silicone
12. History of toxic shock syndrome
13. Currently receiving chemotherapy or immunosuppressive agents
14. Use of investigative drugs within 30 days or 5 half-lives.
15. Currently using or suspected to be using non-therapeutic injection drugs
