# Supplementary material for: Phase I trial of pod-intravaginal rings delivering antiretroviral agents for HIV-1 prevention: Rectal drug exposure from vaginal dosing with tenofovir disoproxil fumarate, emtricitabine, and maraviroc
Source: PLoS One. 2018 Aug 22;13(8):e0201952. doi: 10.1371/journal.pone.0201952 (PMC6104940; doi:10.1371/journal.pone.0201952)
Supplement: S1 Table — (DOCX) [file pone.0201952.s003.docx]

**S1 Table. Drug concentrations in rectal fluid samples collected on the day of TDF pod-IVR removal (six participants); i.e., Day 7.**

|  | **Participant ID** | | | | | |
| --- | --- | --- | --- | --- | --- | --- |
| **Analyte**  **(ng mg^-1^)** | **479-08** | **479-12** | **479-16** | **479-17** | **479-19** | **479-20** |
| TFV | 0.202 | *0.007*^a^ | 0.658 | 0.589 | 0.932 | 0.861 |

^a^BLQ; estimated according to equation 1 (see methods)
