# Supplementary material for: Phase I trial of pod-intravaginal rings delivering antiretroviral agents for HIV-1 prevention: Rectal drug exposure from vaginal dosing with tenofovir disoproxil fumarate, emtricitabine, and maraviroc
Source: PLoS One. 2018 Aug 22;13(8):e0201952. doi: 10.1371/journal.pone.0201952 (PMC6104940; doi:10.1371/journal.pone.0201952)
Supplement: S2 Table — (DOCX) [file pone.0201952.s004.docx]

**S2 Table. Drug concentrations in rectal fluid samples collected on the day of TDF-FTC pod-IVR removal (six participants); i.e., Day 7.**

|  | **Participant ID** | | | | | |
| --- | --- | --- | --- | --- | --- | --- |
| **Analyte**  **(ng mg^-1^)** | **479-08** | **479-12** | **479-16** | **479-17** | **479-19** | **479-20** |
| TFV | 3.505 | 0.158 | 0.019 | 0.958 | 1.269 | 2.444 |
| FTC | 30.502 | 2.710 | 24.235 | 13.155 | 16.343 | 116.541 |
