# Supplementary material for: Phase I trial of pod-intravaginal rings delivering antiretroviral agents for HIV-1 prevention: Rectal drug exposure from vaginal dosing with tenofovir disoproxil fumarate, emtricitabine, and maraviroc
Source: PLoS One. 2018 Aug 22;13(8):e0201952. doi: 10.1371/journal.pone.0201952 (PMC6104940; doi:10.1371/journal.pone.0201952)
Supplement: S3 Table — (DOCX) [file pone.0201952.s005.docx]

**S3 Table. Drug concentrations in rectal fluid samples collected on the day of TDF-FTC-MVC pod-IVR removal (six participants); i.e., Day 7.**

|  | **Participant ID** | | | | | |
| --- | --- | --- | --- | --- | --- | --- |
| **Analyte**  **(ng mg^-1^)** | **479-08** | **479-19** | **479-28** | **479-31** | **479-32** | **479-38** |
| TFV | *0.004*^a^ | *0.004*^a^ | *0.004*^a^ | *0.004*^a^ | 0.035 | *0.004*^a^ |
| FTC | 0.097 | *0.016*^a^ | 0.249 | *0.016*^a^ | 0.419 | 0.117 |
| MVC | 0.653 | 0.212 | 1.120 | 1.031 | 1.736 | 0.119 |

^a^BLQ; estimated according to equation 1 (see methods)
